# Lack of Association Between BsmI and FokI Polymorphisms of the VDR Gene and Sporadic Colorectal Cancer in a Romanian Cohort—A Preliminary Study

**DOI:** 10.3390/curroncol31100476

**Published:** 2024-10-21

**Authors:** Bianca Petre-Mandache, Emilia Burada, Mihai Gabriel Cucu, Diter Atasie, Anca-Lelia Riza, Ioana Streață, Radu Mitruț, Răzvan Pleșea, Amelia Dobrescu, Andrei Pîrvu, Gabriela Popescu-Hobeanu, Paul Mitruț, Florin Burada

**Affiliations:** 1 Doctoral School, University of Medicine and Pharmacy of Craiova, 200349 Craiova, Romania; aknaib86@gmail.com (B.P.-M.); radumitrut@yahoo.co.uk (R.M.); gmph94@gmail.com (G.P.-H.); 2 Laboratory of Human Genomics, University of Medicine and Pharmacy of Craiova, 200638 Craiova, Romania; anca.costache@umfcv.ro (A.-L.R.); ioana.streata@umfcv.ro (I.S.); razvan.plesea@umfcv.ro (R.P.); amelia.dobrescu@umfcv.ro (A.D.); andrei.crgm@gmail.com (A.P.); florin.burada@umfcv.ro (F.B.); 3 Department of Physiology, University of Medicine and Pharmacy of Craiova, 200349 Craiova, Romania; emilia.burada@umfcv.ro; 4 Regional Centre of Medical Genetics Dolj, Emergency Clinical County Hospital Craiova, 200642 Craiova, Romania; 5 Department of Clinical Medicine, Faculty of Medicine, “Lucian Blaga” University, 550024 Sibiu, Romania; 6 Department of Medical Semiology, University of Medicine and Pharmacy of Craiova, 200349 Craiova, Romania; paul.mitrut@umfcv.ro

**Keywords:** colorectal cancer, vitamin D receptor, gene, single nucleotide polymorphism, genotype

## Abstract

Colorectal cancer (CRC) is a major public health problem worldwide, currently ranking third in cancer incidence and second in mortality. Multiple genes and environmental factors have been involved in the complex and multifactorial process of CRC carcinogenesis. VDR is an intracellular hormone receptor expressed in both normal epithelial and cancer colon cells at various levels. Several VDR gene polymorphisms, including FokI and BsmI, have been evaluated for their possible association with CRC susceptibility. The aim of our study was to investigate these two SNPs for the first time in Romanian CRC patients. FokI (rs228570 C>T) and BsmI (rs1544410 A>G) were genotyped by real-time polymerase chain reaction (RT-PCR) in 384-well plates using specific TaqMan predesigned probes on a ViiA™ 7 RT-PCR System. A total of 441 subjects (166 CRC patients and 275 healthy controls) were included. No statistically significant difference was observed between CRC patients and controls when we compared the wild-type genotype with heterozygous and mutant genotypes for both FokI (OR 0.85, 95% CI: 0.56–1.28; OR 0.95, 95% CI: 0.51–1.79, respectively) and BsmI (OR 0.97, 95% CI: 0.63–1.49; OR 1.10, 95% CI: 0.65–1.87, respectively) or in the dominant and recessive models. Also, we compared allele frequencies, and no correlation was found. Moreover, the association between these SNPs and the tumor site, TNM stage, and histological type was examined separately, and there was no statistically significant difference. In conclusion, our study did not show any association between FokI and BsmI SNPs and CRC susceptibility in a Romanian population. Further studies including a larger number of samples are needed to improve our knowledge regarding the influence of VDR polymorphism on CRC susceptibility.

## 1. Introduction

Colorectal cancer (CRC) is a major public health problem, currently ranking third in cancer incidence and second in mortality. Despite revised treatment advances, CRC remains a considerable cause of cancer mortality accounting for 8% of all cancer-related deaths, with an estimated 1.9 million new cancer cases and 935,173 deaths worldwide in 2020 [1,2]. In recent years, the incidence of CRC has grown significantly in Romania, being estimated at 13.1% of all cancers and placing second (12.4%) in terms of mortality rate after lung cancer (19.8%) [3].

Carcinogenesis is a multistep and multifactorial biological process involving complex interactions between multiple genes and environmental factors. This process is actually a series of consecutive mutations and/or epimutations (heritable changes in the epigenetic marks of a gene or a genomic region that led to altered gene expression patterns) leading to uncontrolled cell growth and dysregulation of hedonic homeostatic systems. Oncogenic changes gradually alter cell metabolism, behavior, proliferation control and the way surrounding cells communicate with each other, thus providing an opportunity to evade the immune system. In brief, cancer cells are able to replicate and divide themselves despite genetic and/or epigenetic damage, making cancer a process of uncontrolled cell proliferation [4,5,6]. Despite many advances in genetic research that led to the identification of many associated CRC candidate genes, the specific mechanisms of its development require further research.

The observation of an ecologic correlation between solar radiation exposure and CRC [7] has spawned a number of epidemiologic studies linking dietary vitamin D intake, serum levels of vitamin D metabolites, and vitamin D receptor gene variants with CRC [8]. Vitamin D is known to play an important role in calcium absorption and its ligand (1,25-dihydroxy vitamin D3), the biologically active form of vitamin D, has many extra-skeletal effects such as antiproliferative, anti-apoptotic, and pro-differentiation properties [8,9].

The vitamin D receptor (VDR) is a trans-acting transcriptional regulatory factor that interacts with particular nucleotide sequences of target genes and is encoded by a gene of over 100 kb [10]. The VDR gene is located on chromosome 12q13.11 and consists of five promoters, eight coding exons, and six untranslated exons [11].

During activation, VDR forms a heterodimer with retinoid X receptors, and the result is a complex that binds vitamin D response elements in chromatin regions, thus regulating the expression of many target genes. The active metabolite of vitamin D, 1,25(OH)2D3, regulates vitamin D gene transcription by binding to the vitamin D receptor (VDR) [12,13].

VDR is an intracellular hormone receptor expressed in normal colon epithelial cells and colon cancer (mutated) cells at various levels. VDR downregulation is associated with poor prognosis and cancer progression, while higher expression of VDR in epithelial colon cells correlates with better differentiation and prognosis [8]. Animal studies and human case-control studies provide evidence that vitamin D reduces the risk of CRC and improves prognosis [14,15,16,17,18].

Recently, VDR gene polymorphisms, including FokI, BsmI, ApaI, TaqI, and Tru9I, have been evaluated in genetic association studies of CRC [12,19,20,21,22]. The most frequently studied single nucleotide polymorphisms (SNPs) are the restriction fragment FokI (rs2228570) and BsmI (rs1544410), defined by the endonucleases FokI and BsmI, respectively.

FokI is a type of IIS restriction endonuclease that recognizes a 5 bp asymmetric DNA sequence and cleaves both DNA strands at fixed positions relative to their target sequence [23,24,25,26,27]. It includes two domains connected by a flexible linker as follows: an N-terminal domain, which binds but does not cleave cognate sequences, and a C-terminal domain, which cleaves DNA nonspecifically [22].

The FokI SNP consists of a thymine/cytosine (T/C) substitution in the first of two potential start (ATG) codons and is located in exon 2 of the VDR gene. Thus, the VDR gene from individuals with the T variant, homozygotes for the presence of the restriction site, possesses two potential initiation codons [28]. The F allele is associated with the C variant (wildtype), where the initial ATG is absent, leading to a shorter VDR. On the other hand, the f allele, represented by the T variant, begins at the first ATG (mutant) [29]. This transition changes the location of the start codon (from ATG TO ACG) and produces a three-amino-acid-shorter VDR protein that has higher transcriptional activity [30,31,32].

BsmI is a type II REase class II that cleaves outside of its recognition sites, leading to the designation of the ‘S’ subtype, which refers to ‘shifted cleavage’ [24]. BsmI class II endonuclease can exist as a monomer in both solution and DNA-bound form [33]. The BsmI SNP is located at intron 8 near the 3′ end of the gene. The functional property of this polymorphism is not entirely known, but it may be linked to the microsatellite repeat in the 3′ untranslated region, which affects mRNA stability [34].

The aim of our study was to investigate these two polymorphisms, BsmI and FokI, of the VDR gene for the first time in Romanian patients with sporadic colorectal cancer and to assess the correlation with the risk of CRC.

## 2. Materials and Methods

### 2.1. Subjects

A total of 441 subjects were included in our study. One hundred sixty-six blood samples from patients with sporadic CRC and 275 healthy controls obtained from the Internal Medicine, Oncology, and Gastroenterology departments of the County Emergency Clinical Hospital of Craiova were genotyped for FokI and BsmI SNPs.

Patients were eligible for inclusion in the case group if they were diagnosed with sporadic colorectal adenocarcinoma, confirmed by histopathological findings. All cases were identified as having no prior history of hereditary cancer syndromes, such as familial adenomatous polyposis or hereditary nonpolyposis colorectal cancer.

Patients were excluded from the case group if they had secondary colorectal adenocarcinoma resulting from a primary malignancy located in another organ. Additionally, patients with a known history of any other primary malignancy were also excluded to ensure that this study focused exclusively on sporadic colorectal cancer.

The control group consisted of healthy individuals without any history of colorectal cancer or other malignancies. The controls were matched to the case group by age and gender to ensure comparability between the groups.

This study was conducted in accordance with the Declaration of Helsinki of 1975 (https://www.wma.net/what-we-do/medical-ethics/declaration-of-helsinki/, accessed on 10 January 2019), revised in 2013, and it was approved by the Ethics Committee of the University of Medicine and Pharmacy of Craiova (No. 39/18.02.2019).

### 2.2. SNP Genotyping

Each participant provided a blood sample collected in a 6 mL tube with ethylenediaminetetraacetic acid (EDTA) and stored at 4 °C. Genomic DNA was extracted from peripheral blood leukocytes in a 300 µL sample of whole blood using Wizard^®^ Genomic DNA Purification Kit (Promega, Madison, WI, USA), following the manufacturer’s protocol. After DNA purification, all samples were assessed for quantity and quality, the target values being at least 20 ng of DNA. Upon measuring the contamination via mass spectrophotometry, we targeted at least a 1.7 absorption rate at 260 and 320 nm. However, for a downstream PCR, all samples should have a similar amount of DNA as the starting material; thus, a normalization step was used prior. FokI (rs228570 C>T) and BsmI (rs1544410 A>G) were genotyped by real-time polymerase chain reaction (RT-PCR) in 384-well plates using specific TaqMan predesigned probes. Prior to running all samples, we assessed the amplification rate of both DNA probes with random samples under different conditions. Consequently, each plate included at least two negative controls (no DNA). RT-PCR was performed on a ViiA™ 7 RT-PCR System (Life Technologies, Carlsbad, CA, USA), and SNP genotyping was carried out in a 5 μL reaction volume. The PCR reaction mix volume consisted of 2.5 µL reaction master mix, 1.125 µL ultrapure water, and 0.250 µL probe mix for each sample. To this, 1.25 μL DNA extract was added for a total reaction volume of 5 µL. The reaction’s thermal profile was 60 °C for 30 s, then 95 °C for 10 min, followed by 40 cycles of 95 °C for 15 s, 60 °C for 1 min, and 60 °C for 30 s. The genotype identification was performed blind without information on the subject phenotype. The genotypes were identified by FAM and VIC dye labeling, and an ROX channel was used as a passive reference.

### 2.3. Statistical Analysis

Hardy–Weinberg equilibrium of the SNP genotypes was analyzed by the goodness-of-fit chi-square (*χ*^2^) test. Logistic regression analysis was used to evaluate the association between the VDR gene polymorphisms (FokI and BsmI) and colorectal cancer (CRC) susceptibility. The analysis was adjusted for potential confounding factors, specifically age, and gender, to account for their influence on CRC risk. Odds ratios (ORs) and 95% confidence intervals (CIs) were calculated to quantify the strength of association under different genetic models, including codominant, dominant, recessive, and allelic models.

We evaluated the association between the FokI and BsmI polymorphisms of the VDR gene and colorectal cancer (CRC) risk using several genetic models. The codominant model assesses the risk of CRC across each genotype separately (wild-type, heterozygous, and mutant); the dominant model compares individuals carrying at least one mutant allele (heterozygous and mutant) against those with the wild-type genotype; the recessive model compares those with two mutant alleles to those with one or none; and the allelic model examines the frequency of the mutant versus wild-type allele in cases and controls. These models provided a comprehensive analysis of potential associations between these SNPs and CRC under different genetic inheritance patterns.

The statistical analysis was performed using Microsoft Excel 2019 MSO (Version 2409) software and SPSS Statistics for Windows, Version 22.0 (IBM SPSS Statistics for Windows, Version 22.0, Armonk, NY, USA).

To compare the baseline characteristics between the case and control groups, we used different statistical tests based on the type of data. For categorical variables, such as gender distribution, *p*-values were calculated using the chi-square (χ^2^) test. This test was used to assess whether the distribution of categorical variables significantly differed between the case and control groups. For continuous variables, such as age, *p*-values were calculated using the independent *t*-test. This test was employed to compare the means of continuous variables between the two groups.

The minor allele frequency (MAF) was calculated for each SNP in both the case and control groups. MAF represents the frequency of the less common allele in the population and is a key measure in genetic studies as it helps to understand the distribution of genetic variants. The MAF results provide insights into the genetic diversity of the study population and are critical for comparing allele frequencies between cases and controls.

## 3. Results

**Figure 1:**
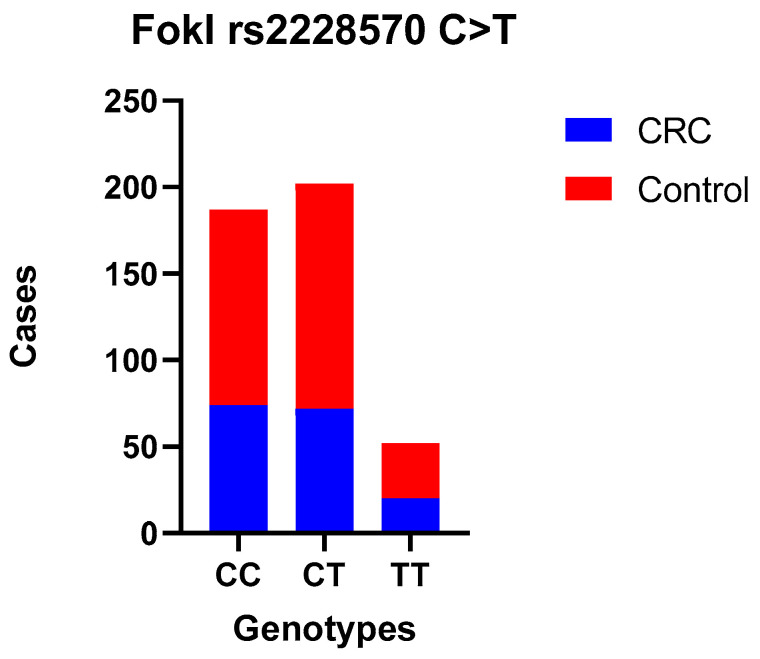
FokI polymorphism—TT, CC, and CT genotype codominant model.

**Figure 2:**
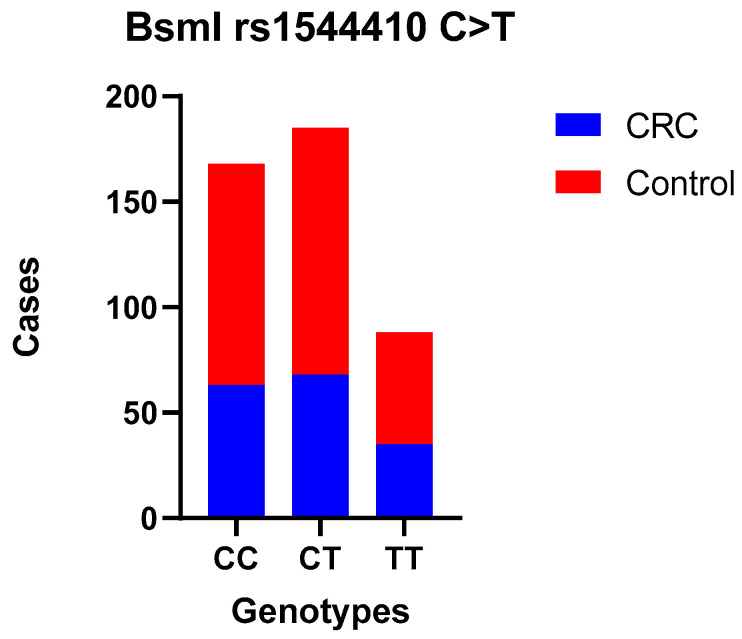
BsmI polymorphism—TT, CC, and CT genotype codominant model.

The main characteristics of the subjects in the case and control groups are summarized in Table 1. Cases and controls were matched in terms of gender and age, and no significant differences were observed between the two groups (*p* > 0.05). The CRC tumor site was proximal, distal, and rectal in 27, 71, and 68 cases, respectively. Among case and controls, genotype distributions were in Hardy–Weinberg equilibrium for both assessed SNPs (χ^2^ = 0.34, *p* = 0.56 control and χ^2^ = 0.45, *p* = 0.50 case group for rs2228570 FokI and χ^2^ = 3.79, *p* = 0.051 control and χ^2^ = 0.41, *p* = 0.05 case group for rs1544410 BsmI) and are presented in Table 2 and Table 3. Table 4 presents the frequencies of each genotype and the association between rs2228570 FokI and rs1544410 BsmI SNPs and the risk of CRC under multiple models of inheritance. The codominant model of inheritance for both rs2228570 FokI and rs1544410 BsmI SNPs can be seen in Figure 1 and Figure 2.

No statistically significant difference was observed between CRC patients and controls when comparing the wild-type genotype with the heterozygous (OR = 0.85 (0.56–1.28)) and mutant genotypes (codominant models—the most common genotype serves as reference OR = 0.95 (0.51–1.79)) for FokI; heterozygous (OR = 0.97 (0.63–1.49)) and mutant genotypes (OR = 1.10 (0.65–1.87)) for BsmI; or when we used dominant and recessive models. Also, we compared allele frequencies, and no correlation was found. Moreover, the association between these SNPs and the tumor site, TNM stage, and histological grade was examined separately, and there was no statistically significant difference (Table 5, Table 6, Table 7 and Table 8).

### Post Hoc Power Analysis

To assess the robustness of our findings, a post hoc power analysis was conducted based on the observed effect sizes in our study. The analysis revealed that with the current sample size of 166 CRC patients and 275 healthy controls, our study had approximately 80% power to detect an effect size of 0.30 at a significance level of 0.05.

## 4. Discussion

Undoubtedly, the risk of sporadic CRC has been linked to environmental conditions (e.g., lifestyle, dietary habits, smoking) and genetic factors [35]. Vitamin D insufficiency has been linked to various diseases and metabolic disorders, including cancers [36]. The functionality of vitamin D is primarily dependent on its interaction with the VDR gene. In cases where the VDR gene exhibits reduced responsiveness, this can result in a manifestation of vitamin D deficiency. Additionally, the VDR gene plays a crucial role in innate immunity, contributing to the prevention and eradication of infections and influencing the composition of the gut microbiome [37,38,39].

Research indicates a notable downregulation of VDR (Vitamin D Receptor) expression in the progression of CRC, with studies highlighting that high levels of VDR are associated with a better prognosis in CRC cases) [10,40].

The VDR gene hosts over 60 single nucleotide polymorphisms (SNPs), prominently featuring the FokI polymorphism at exon 2′s 5′ end and encompasses other polymorphisms, such as BsmI, ApaI, Tru9I (located in intron 8), and TaqI (in exon 9), all situated near the 3′ untranslated region (UTR) of the FokI gene and the CDX-2, an intestinal-specific transcription factor located in the VDR’s 5′ region. Genetic variations in the VDR gene (FokI, BsmI, ApaI, TaqI, Cdx^−2^, Tru9I) have been significantly linked to several types of cancer including prostate, breast, skin, bladder, ovarian, hepatocellular carcinoma, and colorectal cancer [12,21,36,41,42,43,44].

However, only a few are potentially functional and affect the expression of the VDR gene in relation to CRC risk. These include TaqI, located in exon 9, ApaI and BsmI, located in the intron between exons 8 and 9, and FokI, located in exon 2 [21,45,46,47].

The selection of the investigated SNPs in the VDR gene, rs1544410 (BsmI) and rs2228570 (FokI) was based on previous studies and their influence on gene expression.

The FokI polymorphism results in variation at the translation start site, leading to the following protein isoforms: the shorter F-VDR, which exhibits higher transcriptional activity, and the longer f-VDR. This difference in transcriptional activity is thought to be due to the altered interaction between these isoforms and transcriptional machinery and corepressors, ultimately influencing the expression of VDR target genes [2,31,48].

The BsmI polymorphism, located near the 3′ end of the gene, may impact VDR mRNA stability or splicing efficiency, potentially affecting the levels of VDR protein produced. Although the exact functional consequences of BsmI are less well-characterized, its location suggests a role in post-transcriptional regulation [48,49].

In our study, we found no correlation between the investigated SNPs, FokI (rs228570 C>T) and BsmI (rs1544410 A>G), and CRC susceptibility in any comparison model or stratified analysis by histological grade or cancer site in the Romanian population. Our analysis revealed that both FokI and BsmI SNPs were in Hardy–Weinberg equilibrium in both the case and control groups, indicating that the allele frequencies are stable in the population studied. The minor allele frequency (MAF) values were similar between the cases and controls, suggesting no substantial difference in allele frequency distribution between the groups. This consistency further supports the lack of association between these SNPs and CRC susceptibility in the study group. The lack of association between these SNPs does not rule out the possibility of the involvement of other VDR SNPs in the development of CRC.

Our results are in agreement with several other studies considering FokI polymorphism. The findings of Laczmanska et al. showed no correlation between the FokI polymorphism and CRC, and there was no evidence of linkage disequilibrium with other genetic loci [45]. No correlation between FokI genotypes or alleles and susceptibility to CRC was observed by Latacz et al. [22].

Alkhayal et al. revealed that none of the SNPs they studied demonstrated a significant association with CRC in a comprehensive analysis, considering both genotypic and allelic models [50]. In a case-control study conducted by Cho et al., no significant association with CRC risk was found, but a borderline significant association for a lower risk of colon cancer in heterozygous carriers was observed. The same study also noted an increased risk of inflammatory bowel disease in carriers of the f allele [46,51]; our study found no correlation between FokI polymorphism and CRC susceptibility. In a large meta-analysis by Bai et al., no significant association between FokI and CRC risk in the overall and subgroup analyses was found [21].

In their study of 397 subjects from the Greek population, Messaritakis et al. observed a correlation, but it was restricted to KRAS mutations. Notably, the ff genotype occurred more commonly in patients with KRAS mutations, while the Ff and FF genotypes were predominantly seen in patients without KRAS mutations [52]. Sarkissyan et al. found that the FF genotype of FokI was a statistically significant polymorphism associated with colorectal cancer, but their CRC cohort included only 78 patients [46]. Another two studies conducted on the Thai and Iranian populations did not reveal any association between FokI and CRC [53,54]. Furthermore, three different meta-analyses did not reveal associations between VDR FokI polymorphism and CRC risk in any genetic model [12,34,43]. Pan et al. found that FokI might be a risk factor for CRC, the polymorphism being on the fringe of statistically significant in the comparisons of F allele vs. f allele in a fixed model [50,55].

Our results are similar to other studies and meta-analyses in which no correlation was observed in different populations, including Iranians [54], Brazilians [56], Thai [53], and Japanese [57]. Moreover, in their meta-analysis, Touvier et al. observed no correlation when excluding studies with high heterogeneity [34].

On the other hand, our BsmI findings were not consistent with other studies and meta-analyses. Yu et al. suggested that the BB genotype variant of the BsmI gene is linked to a reduced risk of CRC compared with the wild-type bb homozygote, and this reduced risk is also observed in both dominant and recessive genetic models [58]. Similarly Xu et al. observed associations in the homozygote model comparing bb versus BB, in the dominant model comparing bb + Bb versus BB, and in the recessive model comparing bb versus BB + Bb [43]. Pan et al. noted significant differences in allele frequencies, as well as in the homozygous and dominant models, when comparing CRC patients with healthy controls [55]. Raimondi et al. indicated a notable decrease in the risk of CRC for individuals with the BsmI BB, Bb, and Bb + BB genotypes in comparison with those with the bb genotype; additionally, a tendency towards decreased cancer risk associated with the presence of the B allele was reported [59]. Bai et al. found that individuals with the BB or Bb genotypes were associated with a significant decrease in CRC risk compared with patients carrying the bb genotype. The dominant model (BB + Bb vs. bb) and the recessive model (BB vs. Bb + bb) also showed a significant association with CRC risk. In addition, the BB genotype showed a decreased risk for colon cancer compared with the bb or Bb + bb genotypes. However, no significant differences were observed between this SNP and rectal cancer risk [21].

### Limitations

Our study has certain limitations that need to be considered. Firstly, the sample size is relatively small.

The sample size in this study may not have been large enough to identify smaller but potentially meaningful associations between SNPs in VDR genes and CRC. This limitation is particularly important when stratifying by tumor site, stage, or other subgroups, which reduces the effective sample size and statistical power. To address this limitation, future studies should aim to include larger sample sizes or aggregate data through meta-analyses, which would enhance the ability to detect smaller effect sizes and provide more robust conclusions. These approaches would help validate our findings and potentially uncover subtle genetic influences on CRC susceptibility that were not apparent in our study.

Secondly, we only assessed two SNPs. Additionally, there is a lack of functional analysis, gene expression data, and clinical and biochemical data such as serum levels of vitamin D. Future research should broaden the scope to include a wider range of SNPs within the VDR gene, as well as in the related pathways, to gain a more comprehensive understanding of the genetic factors involved in CRC. Additionally, it is important to consider gene–gene and gene–environment interactions, which may play a critical role in modulating the risk of CRC. Investigating these interactions could provide valuable insights into the complex network of factors contributing to CRC development and could lead to more targeted prevention and treatment strategies. Thirdly, the potential for confounding factors and biases should be considered. Although we adjusted for age and gender in our analyses, the presence of additional uncollected confounding factors may have influenced our results. Variables such as dietary habits, levels of physical activity, and exposure to sunlight, which can impact vitamin D levels, were not factored into our analyses and may have affected the outcomes of our study. Moreover, the control group was selected to be cancer-free, but differences in lifestyle or environmental exposures between cases and controls could introduce bias. To minimize these issues, future studies should collect detailed data on potential confounders and consider using more sophisticated statistical methods, such as multivariable regression models or propensity score matching, to account for these factors.

One plausible explanation for our results could be the potential connection of these SNPs to yet-undiscovered genetic variants, the size of cohorts, and population heterogeneity. Additionally, epistatic interactions (where the effect of one gene is modified by another) could obscure simple associations. Also, environmental and lifestyle factors, such as dietary (e.g., calcium and vitamin D intake) and physical activity, have been shown to influence expression levels in conjunction with VDR gene polymorphisms [57,60,61].

To better define the importance of VDR genetic variants in association with CRC, assessments of other variants are needed, as well as functional studies.

## 5. Conclusions

In conclusion, our study did not show any association between FokI and BsmI SNPs and CRC susceptibility in a Romanian population. However, while our findings suggest that these particular polymorphisms may not be significant risk factors for CRC in this cohort, future research should explore the broader implications of VDR polymorphisms, particularly in relation to cancer progression, recurrence, survival, and drug resistance. Given the role of vitamin D in cell proliferation, immune response, and drug metabolism, VDR polymorphisms may influence not only the risk of CRC but also its clinical outcomes. Further studies with larger sample sizes are needed to enhance our understanding of the influence of VDR polymorphisms on CRC susceptibility and to investigate their potential impact on patient prognosis and treatment responses. This could lead to more personalized approaches to managing CRC, ultimately improving patient outcomes.

## Figures and Tables

**Table 1:** Subject characteristics.

Variable	Colorectal Cancer	Control	*p* Value
Number. of cases	166	275	
Male/Female	108/58	176/99	0.357 *
Age (years), mean ± SD	65 ± 9.65	59 ± 11.6	0.33 *
Location			
− Proximal.	27		
− Distal.	71		
− Rectum.	68		
Tumor stage—Dukes’ stage			
− A + B	115		
− C + D	51		
Differentiation grade			
− G1—well.	55		
− G2—moderate.	95		
− G3—poor.	16		

* chi-square (χ^2^) test.

**Table 2:** Minor allele frequencies and Hardy–Weinberg equilibrium values in the control group.

SNP	MAF *	χ^2^	*p*
FokI rs2228570 C>T	0.35	0.34	0.56
BsmI rs1544410 C>T	0.41	3.79	0.051

* MAF—minor allele frequency.

**Table 3:** Minor allele frequencies and Hardy–Weinberg equilibrium values in case group.

SNP	MAF *	χ^2^	*p*
FokI rs2228570 C>T	0.35	0.45	0.50
BsmI rs1544410 C>T	0.41	3.95	0.05

* MAF—minor allele frequency.

**Table 4:** Association between FokI and BsmI SNPs and CRC risk under multiple models of inheritance.

SNP	CRC (n = 166)	Control (n = 275)	OR (95%CI)	*p* Value
**FokI rs2228570 C>T**				
*Codominant*				
CC (FF)	74 (44.58%)	113 (41.09%)	Reference	-
CT (Ff)	72 (43.37%)	130 (47.27%)	0.85 (0.56–1.28)	0.42
TT (ff)	20 (12.05%)	32 (11.64%)	0.95 (0.51–1.79)	0.89
*Dominant*				
CC	74 (44.58%)	113 (41.09%)	Reference	-
CT + TT	92 (55.42%)	162 (58.91%)	0.87 (0.59–1.28)	0.47
*Recessive*				
CC (FF) + CT (Ff)	146 (87.95%)	243 (88.36%)	Reference	-
TT (ff)	20 (12.05%)	32 (11.64%)	1.04 (0.57–1.89)	0.90
*Allelic*				
C (F)	220 (66.27%)	356 (64.73%)	Reference	-
T (f)	112 (33.73%)	194 (35.27%)	0.93 (0.70–1.24)	0.64
**BsmI rs1544410 C>T**				
*Codominant*				
CC (BB)	63 (37.95%)	105 (38.18%)	Reference	-
CT (Bb)	68 (40.96%)	117 (42.55%)	0.97 (0.63–1.49)	0.88
TT (bb)	35 (21.09%)	53 (19.27%)	1.10 (0.65–1.87)	0.72
*Dominant*				
CC (BB)	63 (37.95%)	105 (38.18%)	Reference	-
CT (Bb) + TT (bb)	103 (62.05%)	170 (61.82%)	1.01 (0.68–1.50)	0.96
*Recessive*				
CC (BB) + CT (Bb)	131 (78.91%)	222 (80.73%)	Reference	-
TT (bb)	35 (21.09%)	53 (19.27%)	1.12 (0.69–1.81)	0.65
*Allelic*				
C (B)	194 (58.43%)	327 (59.45%)	Reference	-
T (b)	138 (41.57%)	223 (40.55%)	1.04 (0.79–1.38)	0.76

**Table 5:** Association between FokI and BsmI polymorphisms and colorectal cancer in the tumor site subgroups.

Polymorphism	Proximal CRC		Distal CRC		Rectal CRC	
	N = 27	OR (95%CI); *p*	N = 71	OR (95%CI); *p*	N = 68	OR (95%CI); *p*
**rs2228570 C>T**						
**(FokI)**						
CC	10	Reference	28	Reference	36	Reference
CT	15	1.30 (0.56–3.01); 0.53	30	0.93 (0.53–1.65); 0.81	27	0.65 (0.37–1.14); 0.13
TT	2	0.71 (0.15–3.39); 0.65	13	1.64 (0.76–3.53); 0.21	5	0.49 (0.18–1.35); 0.14
T carriers	17	1.19 (0.52–2.69); 0.49	43	1.07 (0.63–1.83); 0.80	32	0.62 (0.36–1.06); 0.08
**rs1544410 C>T**						
**(BsmI)**						
CC	11	Reference	24	Reference	28	Reference
CT	8	0.65 (0.25–1.68); 0.37	30	1.12 (0.62–2.04); 0.71	30	0.96 (0.54–1.71); 0.89
TT	8	1.44 (0.55–3.79); 0.46	17	1.40 (0.69–2.84); 0.35	10	0.71 (0.32–1.56); 0.38
T carriers	16	0.90 (0.40–2.01); 0.79	47	1.21 (0.69–2.09); 0.49	40	0.88 (0.51–1.51); 0.65

**Table 6:** Association between FokI and BsmI polymorphisms and risk of colon and rectal cancer.

Polymorphism	N = 98	CRC OR (95%CI); *p*	N = 68	Rectal OR (95%CI); *p*
**rs2228570 C>T**				
**(FokI)**				
C	38	Reference	36	Reference
CT	45	1.03 (0.62–1.70); 0.91	27	0.65 (0.37–1.14); 0.13
TT	15	1.39 (0.68–2.85); 0.37	5	0.49 (0.18–1.35); 0.14
T carriers	60	1.10 (0.69–1.77); 0.69	32	0.62 (0.36–1.06); 0.08
**rs1544410 C>T**				
**(BsmI)**				
CC	35	Reference	28	Reference
CT	38	0.97 (0.57–1.65); 0.92	30	0.96 (0.54–1.71); 0.89
TT	25	1.41 (0.77–2.61); 0.27	10	0.71 (0.32–1.56); 0.38
T carriers	63	1.11 (0.69–1.79); 0.66	40	0.88 (0.51–1.51); 0.65

**Table 7:** Association between FokI and BsmI polymorphisms and TNM stage of colorectal cancer.

Polymorphism	Tumor Stage I + II (A + B) n = 115 (%)	OR (95%CI); *p*	Tumor Stage III + IV (C + D) n = 51 (n %)	OR (95%CI); *p*
**rs2228570 C>T**				
**(FokI)**				
CC (FF)	58 (50.44%)	Reference	16 (31.37%)	Reference
CT (Ff)	45 (39.13%)	0.67 (0.42–1.07); 0.09	27 (52.94%)	1.47 (0.75–2.86); 0.26
TT (ff)	12 (10.43%)	0.73 (0.35–1.52); 0.40	8 (15.69%)	1.77 (0.69–4.50); 0.24
T carriers	57 (49.57%)	0.68 (0.44–1.06); 0.09	35 (68.83%)	1.53 (0.81–2.89); 0.19
**rs1544410 C>T**				
**(BsmI)**				
CC	44 (38.26%)	Reference	19 (37.25%)	Reference
CT	44 (38.26%)	0.89 (0.55–1.47); 0.67	24 (47.06%)	1.13 (0.59–2.19); 0.71
TT	27 (23.48%)	1.22 (0.68–2.18); 0.51	8 (15.69%)	0.83 (0.34–2.03); 0.69
T carriers	71 (61.74%)	0.99 (0.64–1.56); 0.99	32 (62.75%)	1.04 (0.56–1.93); 0.90

**Table 8:** Association between FokI and BsmI polymorphisms and colorectal cancer in the histologic grade subgroups.

Polymorphism	G1		G2		G3	
	N = 55	OR (95%CI); *p*	N = 95	OR (95%CI); *p*	N = 16	OR (95%CI); *p*
**rs2228570 C>T**						
**(FokI)**						
CC (FF)	22	Reference	45	Reference	7	Reference
CT(Ff)	27	1.07 (0.58–1.98); 0.84	38	0.73 (0.44–1.21); 0.22	7	0.87 (0.30–2.55); 0.80
TT(ff)	6	0.96 (0.36–2.58); 0.94	12	0.94 (0.45–1.99); 0.87	2	1.01 (0.20–5.09); 0.99
T carriers	33	1.05 (0.58–1.89); 0.88	50	0.77 (0.48–1.24); 0.29	9	0.89 (0.32–2.48); 0.83
**rs1544410 C>T**						
**(BsmI)**						
CC	20	Reference	36	Reference	7	Reference
CT	22	0.98 (0.51–1.91); 0.97	40	0.99 (0.59–1.68); 0.99	5	0.64 (0.19–2.08); 0.46
TT	13	1.29 (0.60–2.79); 0.52	19	1.05 (0.55–1.99); 0.89	4	1.13 (0.32–4.04); 0.85
T carriers	34	1.08 (0.59–1.97); 0.80	60	1.01 (0.63–1.64); 0.96	9	0.79 (0.29–2.19); 0.66

## Data Availability

All data presented here are available from the authors upon reasonable request.
